# Molecular Genetic Understanding of Photoperiodic Regulation of Flowering Time in *Arabidopsis* and Soybean

**DOI:** 10.3390/ijms23010466

**Published:** 2021-12-31

**Authors:** Xiao Luo, Mengnan Yin, Yuehui He

**Affiliations:** 1 Peking University Institute of Advanced Agricultural Sciences, Weifang 261325, China; 2 Chinese Academy of Sciences Center for Excellence in Molecular Plant Sciences, Shanghai 201602, China; mnyin@psc.ac.cn; 3 State Key Laboratory of Protein and Plant Gene Research, Peking-Tsinghua Center for Life Sciences, School of Advanced Agriculture Sciences, Peking University, Beijing 100871, China

**Keywords:** flowering time, photoperiod pathway, FT, florigen, chromatin modification, long day, short day, soybean, *Arabidopsis*

## Abstract

The developmental switch from a vegetative phase to reproduction (flowering) is essential for reproduction success in flowering plants, and the timing of the floral transition is regulated by various environmental factors, among which seasonal day-length changes play a critical role to induce flowering at a season favorable for seed production. The photoperiod pathways are well known to regulate flowering time in diverse plants. Here, we summarize recent progresses on molecular mechanisms underlying the photoperiod control of flowering in the long-day plant Arabidopsis as well as the short-day plant soybean; furthermore, the conservation and diversification of photoperiodic regulation of flowering in these two species are discussed.

In plant life cycles, the time of the trantion to flowering is critical to reproductive success and is regulated by external environmental factors (such as light, temperature) and endogenous signals such as age and developmental state. Seasonal changes in day length (photoperiod) play an important role in the regulation of flowering in various plants, through the photoperiod pathway [1]. Photoperiodic flowering responses are classified into three major types: long-day (LD), short-day (SD), and day-neutral (DN), based on their flowering responses to day-length changes [2,3]. Here, we review recent molecular genetic understandings of how seasonal day-length changes, through the photoperiod pathways, promote flowering in the LD plant *Arabidopsis thaliana* and the SD plant soybean.

## 1. Molecular Mechanisms for the Photoperiodic Regulation of Flowering in *Arabidopsis*

*Arabidopsis thaliana* is a typical LD plant, which flowers when the day length becomes longer, that is, longer-day lengths promote flowering, while shorter-daylengths inhibit flowering in *Arabidopsis*.

### 1.1. The Photoperiod Pathway in Arabidopsis

The photoperiod pathway begins with the perception of light signals by photoreceptors in leaves. Light signals of different wavelengths are received and recognized by the photoreceptors in leaves. Currently, there are three main types of photoreceptors found in plants: phytochromes, cryptochromes and phototropins. Phytochromes mainly absorb red light and far-red light. *Arabidopsis* has at least five types of phytochromes: PHYA, PHYB, PHYC, PHYD and PHYE. PHYA promotes flowering, whereas PHYB, PHYD and PHYE inhibit flowering. PHYC inhibits flowering under SD but promotes flowering with PHYA under LD [4,5,6]. Cryptochromes mainly absorb blue and ultraviolet light, and *Arabidopsis* contains CRY1 (CRYPTOCHROME 1), CRY2 and CRY3. Both CRY1 and CRY2 promote flowering [7]; it is not clear whether CRY3 is involved in the regulation of flowering. So far, it has not been found that phototropins are involved in flowering regulation.

After sensing the photoperiod, photoreceptors transmit the signal to the circadian clock. The clock components such as ELF3 (EARLY FLOWERING 3) [8,9], ELF4 [10,11], PIF3 (PHYTOCHROME INTERACTING FACTOR 3) [12], ZTL (ZEITLUPE) [13], FKF1 (FLAVIN-BINDING KELCH REPEAT F-BOX 1) [13] and DET1 (DEETIOLATED 1) [14,15], have been found to mediate the optical signal input of different photoreceptors. ELF3 encodes a nuclear protein that plays a role in transcriptional regulation, both its mRNA and protein exhibit circadian rhythm [16]. The *elf4* mutant also exhibits disruption of biological circadian with an early flowering phenotype under non-induced photoperiod conditions [10,11], and ELF4 may also regulate the input of light signals [11]. ZTL, FKF1 and LKP2 (LOV KELCH PROTEIN2) regulate biological rhythms and flowering time, and mutations in these genes lead to disruptions of circadian rhythms [17,18,19].

*Arabidopsis thaliana* has a negative feedback regulating loop for maintaining and resetting the circadian clock, which is mainly composed of CCA1 (CIRCADIAN CLOCK ASSOCIATED 1), LHY (LATE ELONGATED HYPOCOTYL) and APRRs (ARABIDOPSIS PSEUDO-RESPONSE REGULATOR 1) [17,20]. LHY and CCA1 belong to the MYB transcription factor family. APRRs include APRR9, APRR7, APRR5, APRR3, APRR1/TOC1 (TIMING OF CAB OF EXPRESSION 1) and other homologous proteins; the expression levels and protein levels of these genes all exhibit circadian rhythm [21]. *LHY* and *CCA1* are activated by light in the morning, and the newly synthesized LHY and CCA1 bind to the *TOC1* promoter and inhibit its expression [22,23,24]. At the same time, blue light promotes the interaction between ZTL and GI (GIGANTEA) and degrades TOC1 through the ubiquitin-protease system [25]. Therefore, at the beginning of the day, LHY and CCA1 gradually increase, and TOC1 gradually decreases [24]. Interestingly, *TOC1* is a necessary promoter for the expression of *LHY* and *CCA1*; therefore, inhibition of *TOC1* will result in down-regulation of the expression of *LHY* and *CCA1* [24]. The levels of *LHY* and *CCA1* drop to a minimum at night, and thus the inhibition of *TOC1* expression is released, resulting in that the expression of *LHY* and *CCA1* is started again, entering a new cycle [24].

The important genes in output of the circadian clock signal include *CO* (*CONSTANS*), *GI* and *FT* (*FLOWERING LOCUS T*). *CO* encodes a transcription factor with a B-box zinc finger structure, and its expression is regulated by the circadian clock with a 24-hour periodic oscillation [26]. The transcription level of *CO* is regulated by FKF1, GI, CDFs (CYCLING DOF FACTOR), and FBH (FLOWERING BHLH). CDFs are plant-specific transcription factors and bind to the regulatory region of *CO* to inhibit its transcription [27]. Under LD, in the morning, *CDF* expression is activated by LHY and CCA1, and when GI accumulates to a certain amount, a CDF1-GI complex is formed to inhibit *CO* transcription [28,29]. After about 13 hours of light, the protein levels of FKF1 and GI reach peaks and these proteins form a dimeric FKF1-GI E3 ubiquitin ligase complex. FKF1-GI targets CDF proteins for degradation by the proteasome, thereby releasing CDF-mediated *CO* inhibition [28,30]. Subsequently, four basic helix-loop-helix (bHLH) transcription factors (TFs) FBH1-FBH4, bind to the E-box *cis*-element to promote *CO* expression [31]. However, under SD, the protein levels of GI and FKF1 reached their peaks at 7 and 10 hours of light, respectively, and are unable to form the FKF1-GI complex, and thus *CO* transcription stays at a low level [28,30]. Therefore, the interaction of FKF1, GI and CDFs leads to higher *CO* transcription levels under LD than in SD.

Posttranslational regulation is very important for CO protein stability. CO protein is degraded in the morning and stabilized near dusk [32]. The red light photoreceptor PHYA and far-red light photoreceptor PHYB antagonistically regulate the stability of CO protein. In the morning, PHYB absorbs red light and interact with the RING finger-containing E3 ubiquitin ligase HOS1 (HIGH EXPRESSION OF OSMOTICALLY RESPONSIVE GENE 1) to promote degradation of the CO protein [32,33]. At night, members of *Arabidopsis* SPA (SUPPRESSOR OF PHYA-105) family interact with another RING-finger E3 ubiquitin ligase COP1 (CONSTITUTIVE PHOTOMORPHOGENIC 1), and this complex binds to CO through a WD-repeat domain, and promotes CO degradation [34,35]. In late afternoon in LDs, red light photoreceptor Phytochrome A (PHYA) and blue light photoreceptors CRY1 and CRY2, all function to stabilize the CO protein [36]. Blue light induces CRY2 phosphorylation, and the phosphorylated CRY2 enhances the CRY2-SPAs interaction to inhibit the function of COP1-SPAs in CO degradation [37]. The blue light also enhances FKF1 stability, which accumulates from the afternoon to dusk and interacts with CO through its LOV domain to stabilize the CO protein [38,39]. PHL (PHYTOCHROME-DEPENDENT LATE FLOWERING), whose expression level is increased in the afternoon, interacts with PHYB and suppresses PHYB-mediated degradation of the CO protein [40]. In addition, DNF (DAY NEUTRAL FLOWERING), a membrane-bound E3 ligase, regulates *CO* in a way independent of GI/FKF1/CDF. The *dnf m*utant exhibits an early-flowering phenotype due to disruption of the circadian rhythm of CO expression [41]. The results of CO regulation by the above factors are: under LD, the CO protein accumulates at dusk; under SD, CO cannot be stably produced and accumulated.

### 1.2. Regulation of FT Expression

The photoperiod pathway output *CO* promotes *FT* expression [42]. The FT protein belongs to the PEBP (phosphatidylethanolamine binding) family and is a major florigen [43,44]. FT moves to SAM (shoot apical meristem) from leaves through the phloem and subsequently interacts with 14-3-3 proteins and the bZIP transcription factor FD (FLOWERING D) to form a transcription-activation complex that promotes the expression of floral meristem-identity genes in SAM [45]. Therefore, *FT* functions as the key floral promoter.

**Figure 1:**
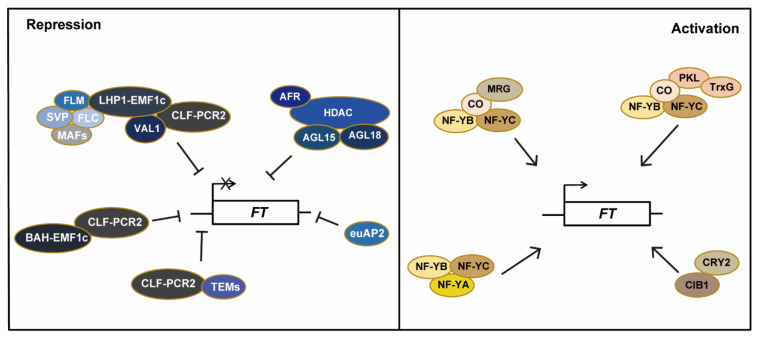
Positive and negative factors are involved in *FT* regulation in *Arabidopsis* under long day (LD). Under LD conditions, LHP1-EMF1c and BAH-EMF1c, together with CLF-PRC2, act to inhibit the expression of *FT* by promoting H3K27me3 deposition and Polycomb repression on *FT* chromatin. The DNA-binding protein VAL1 recruits LHP1-EMF1c to *FT* chromatin. In addition, AFR-HDAC, together with AGL15 and AGL18, downregulate *FT* expression around dusk. SVP, FLC and MAFs repress *FT* expression by recruiting EMF1c; in addition, TEM1 and TEM2 directly bind to the *FT* promoter and repress its expression. euAP2 family TFs, including AP2 (APETALA 2) and three TARGET OF EAT (TOE) proteins (TOE1, TOE2, and TOE3), repress *FT* expression, thereby delaying flowering. PICKLE, a chromatin remodeling factor, antagonizes Polycomb binding to *FT* chromatin with the assistance of the NF-Y complex, resulting in *FT* de-repression and thereby promoting flowering in LDs. TrxG proteins is recruited by PKL to *FT* chromatin to mediate transcriptional activation of *FT* under LDs; in addition, MRG1 (MORF RELATED GENE 1) and MRG2 function to promote H3K4me3 and H3K36me3 on *FT* chromatin specifically around dusk. Lastly, CIB1 interacts with CRY2 to promote *FT* expression.

*FT* expression is regulated by various transcriptional activators and repressors. Several types of transcription inhibitors are involved in the regulation of *FT* expression. From early morning to late afternoon and at night, *FT* chromatin is marked predominantly with repressive histone 3 lysine-27 trimethylation (H3K27me3), so *FT* expression is mainly repressed by Polycomb group (PcG) genes. The H3K27me3 reader LHP1 (LIKE HETEROCHROMATIN PROTEIN 1) and histone H3 lysine-4 demethylase JMJ14 interact with the plant-unique EMF1 (EMBRYONIC FLOWER1) to form a distinct Polycomb repressive complex 1 (PRC1)-like complex called LHP1-EMF1c that represses *FT* expression [46]. In addition, two BAH domain H3K27me3 readers EBS and SHL interact with EMF1 to form the BAH-EMF1c complexes [47,48]. These PRC1-like EMF1c complexes (LHP1-EMF1c and BAH-EMF1c) bind to *FT* chromatin, read and further maintain the H3K27me3 repression marks at *FT*, which are catalyzed by the H3K27 methyltransferase complex, CLF-PRC2 (CURLY LEAF-Polycomb repressive complex 2) (Figure 1).

The RAV subfamily TF TEM1 (TEMPRANILLO 1) and TEM2 function as flowering repressors. TEM1/2 regulate the juvenile-to-adult growth transition and bind to the *FT* promoter to repress *FT* expression [49,50]. Recently, the structural basis of how the DNA-binding domains AP2 and B3 in TEM1 recognize CAACA and CACCTG motifs in the 5′-UTR of *FT*, respectively, has been revealed [51]. The combination of the AP2 and B3 binding sites significantly enhances the binding of TEM1 to *FT* 5′-UTR. The ability of TEM1 AP2 and B3 domains simultaneously binding to *FT* is necessary for recruiting PRC2 and deposition of H3K27me3 in *FT* 5′-UTR, to precisely regulate the floral transition.

Another group of AP2 transcription factors (known as the euAP2 family) also functions to repress *FT* expression. This family consists of AP2 (APETALA 2), the three TOE (TARGET OF EAT) proteins (TOE1, TOE2, and TOE3), and SMZ (SCHLAFMUTZE) and its paralog SNZ (SCHNARCHZAPFEN), which are suppressed by microRNA172 (miR172) [52]. *miRNA172* expression is regulated in a *CO*-independent manner, and *GI* promotes *miRNA172* expression under LD conditions [53]. miR156 regulates the expression of *miR172* by increasing the level of *SPL9* and *SPL10*, which directly promote *miR172* expression in an age-dependent manner [54,55]. SMZ inhibits *FT* expression through directly binding to the *FT* promoter, and therefore, miR156 and miR172 are involved in controlling *SMZ* expression to determine the timing of *FT* expression in a *CO*-independent manner [52]. Under inductive LDs, euAP2 family loci including TOE1, TOE2 and SMZ are also regulated by SIN3 (SWI-INDEPENDENT 3) LIKE proteins, including SIN3 LIKE 1 (SNL1) to SNL5. SNLs bind to euAP2 family loci to mediate histone deacetylation and inhibit their expression, resulting in LD induction of *FT* expression [56]. It is very interesting that under non-inductive SD, SNLs mediate histone deacetylation of the *FT* activator AGL19 and inhibit its expression, thereby indirectly inhibiting *FT* expression, leading to delayed flowering under SD [56].

The MADS box TFs including SVP (SHORT VEGETATIVE PHASE) and the FLC (FLOWERING LOCUS C) family consisting of FLC, FLM (FLOWERING LOCUS M) and MAF2 (MADS AFFECTING FLOWERING 2) to MAF4, functions to repress *FT* expression. SVP physically interacts with FLC to directly repress *FT* expression via binding to the CArG motifs at the *FT* locus [57,58]. In addition, SVP functions together with FLM to mediate *FT* regulation in response to ambient temperature changes [59,60]. The MAF3 protein accumulates at the end of LDs and binds to *FT* chromatin to repress its expression [61]. FLC, FLM, MAF2 and MAF4 interact with SVP, suggesting that these proteins may form a large MADS-domain complexes to repress *FT* expression [60,61].

The level of CO protein is critical for the induction of *FT* expression. The molecular mechanism of the CO protein to regulate *FT* expression through its C-terminal CCT domain has been revealed through structural and biochemical analyses [62]. The N-terminal B-Box domains of CO form a possible tetrameric assembly, and the C-terminal CCT domain of CO interacts with NF–YB/YC (NUCLEAR FACTOR-YB/YC) to form the trimeric CO-CCT-NF-YB/YC complex (NF-CO). Four NF-CO complexes appear to function in concert to specifically bind multiple *cis*-acting TGTG-bearing elements in a proximal *FT* promoter region [62]. In addition, a NF-Y transcription factor complex composed of NF-YA, NF-YB and NF-YC, binds to the CCAAT site located in the distal *FT* promoter, with assistance from the ATPase-dependent chromatin-remodeling factor PKL (PICKLE) [63,64]. NF-CO and NF-Y appear to function together to promote chromatin looping in the *FT* promoter, resulting in a strong reduction of PcG enrichment on *FT* chromatin and consequent *FT* de-repression in leaf veins at dusk [65,66]. *FT* chromatin is also bound by TrxG (Trithorax group) proteins that mediate histone 3 lysine-4 trimethylation (H3K4me3) deposition under LD, and active chromatin modifications are required for *FT* de-repression at around dusk. The TrxG protein H3K4 methyltransferase ATX1 (ARABIDOPSIS HOMOLOG OF TRITHORAX 1) is recruited by PKL to *FT* chromatin specifically at around dusk [67]. In addition, the H3K4me3/H3K36me3 readers MRG1 (MORF RELATED GENE 1) and MRG2 can bind to *FT* chromatin at around dusk in LDs [68,69], to promote H3K4me3 deposition on *FT* chromatin. MRG2 also interacts with the NAP1 (NUCLEOSOME ASSEMBLY PROTEIN 1) family proteins, including NRP1 (NAP1-RELATED PROTEIN 1) and NRP2, and NRP1 and NRP2 inhibit the binding of MRG1/MRG2 to CO, leading to a transcriptional repression of *FT* [70].

*FT* expression is induced by CIB1 (CRYPTOCHROME-INTERACTING BASIC-HELIX-LOOP-HELIX 1), CIB2, CIB4 and CIB5 [71,72,73]. The CIB1 protein is presented from afternoon to early night in LDs, and interacts with CRY2 in a blue light-dependent manner; CIBs bind to the E-box elements (CANNTG) at *FT* to stimulate *FT* expression at around dusk in LDs [73].

Histone acetylation is positively linked with active gene expression in eukaryotes, and the acetylation level is dynamically controlled by histone acetyltransferases (HAT) and histone deacetylases (HDACs) [74]. Histone deacetylation is often associated with gene repression or down-regulation. Upon the activation of *FT* expression near dusk in LDs, HDACs bind to *FT* chromatin at dusk to repress its expression, and thus prevents overproduction of the FT protein. There are two types of HDAC complexes for *FT* repression, including AFR-HDAC and MRG-dependent HD2C complexes [75,76]. AFR-HDAC consists of HDA19 and structural components including a Sin3-like scaffold protein, SAP18 (Sin3-Associated Polypeptide 18), and AFR1 (SAP30 FUNCTION RELATED 1) or AFR2 [75]. AFR-HDAC is recruited to the *FT* locus specifically at the end of LDs by the two MADS-domain transcription factors AGL15 and AGL18, and this recruitment depends on the presence of CO activity. Hence, CO activity not only activates *FT* expression, but also enables the recruitment of an HDAC complex to *FT* to downregulate its expression [75]. HD2C acts as an effective deacetylase, mainly targeting H3K9ac, H3K23ac and H3K27ac. HD2C is recruited to *FT* chromatin to inhibit *FT* transcription at the end of the day in an MRG1/2-dependent manner. HD2C antagonizes CO for the binding of MRG2, and acts to promote the release of CO from *FT* for protein degradation [75]. In the middle of LDs, MRG proteins bind to H3K4me3/H3K36me3-marked chromatin and interact with CO to promote *FT* expression [75]. At dusk, AFR-HDAC and MRG-HD2C may work together to regulate *FT* expression at an appropriate level. This balance finely regulates *FT* expression at an appropriate level, and thus prevents precocious flowering in response to the inductive LD signals.

### 1.3. FT Protein Movement

Under LDs, *FT* mRNA is expressed in CCs (companion cells) of leaf vascular bundles, and the FT protein travels a long distance from leaves to SAM through the phloem to induce flowering. The movement of FT from CCs to sieve elements (SEs) is regulated by FTIP1 (FT-INTERACTING PROTEIN 1), QKY (QUIRKY) and SYP121 (SYNTAXIN OFPLANTS121) [77,78]. FTIP1 is an ER (endoplasmic reticulum) membrane protein, whereas both QKY and FTIP1 belong to the MCTP (multiple C2 domain and transmembrane protein) family. SYP121 is a syntaxin-like Q-SNARE protein with a C-terminal transmembrane domain that can mediate the transport of vesicles to the plasma membrane [79]. The nonfunctional mutants of *ftip1*, *qky* and *syp121* are late-flowering under LDs. FTIP1 is specifically localized in phloem CCs and plasmodesmata between CCs and SEs, and mediates the FT protein transport through the ER system and plasmodesmata [78], SYP121 interacts with QKY, and mediates FT transport to the plasma membrane via endosomal vesicles, thereby promoting FT translocation to SEs [77]. The FT protein export from CCs to SEs is regulated in a temperature-dependent manner, and repressed by low temperature [80].

After FT enters into the phloem stream, the long-distance trafficking of FT protein from SEs to SAM is regulated by NaKR1 (SODIUM POTASSIUM ROOT DEFECTIVE 1), a heavy metal-associated domain-containing protein. Loss of function of *NaKR1* causes late flowering under long-day conditions, largely because of FT transportation to SAM through SEs is hindered [81].

A recent breakthrough indicates that the negatively charged phospholipid PG (phosphatidyl-glycerol) on the cellular membranes of lipid bilayer can interact with the FT protein, and sequester FT [82]. Low temperature promotes FT sequestration in the cellular membrane of the CC, thereby reducing the level of soluble FT and delaying the transition to flowering [82]. These findings reveal the mechanism underlying how plants modulate the activity of florigen to optimize the timing of flowering in response to temperature changes. Low temperature-induced FT protein transport reduction and transcription inhibition act antagonistically to photoperiodic *FT* induction, so as to optimize flowering at a suitable time.

In summary, the molecular circuitry underlying long-day induction of flowering in *Arabidopsis*, composed of photoreceptors, circadian clock, *CO* and *FT*, has been well dissected. As most of the components in this regulatory system are evolutionarily conserved in other flowering plants, molecular understanding of photoperiodic regulation of flowering in *Arabidopsis* has provided the basis for exploring day-length regulation of flowering in other plants such as soybean.

## 2. Molecular Mechanisms for Photoperiodic Regulation of Flowering Time in Soybean

Soybean [*Glycine max (L.) Merr.*] is the main source of human vegetable oil and vegetable protein. Soybean is a typical SD crop, that is, flowering much more earlier in SDs (less than 12 hours) than under the conditions of long day (more than 16 hours). Originated in the 30°～45° north latitude region of China, soybean is grown in a wide range of latitudes and cultivated in broad regions, ranging from 50°N latitude to 35°South latitude. However, cultivation of individual varieties is usually limited to a narrow range of latitudes, mainly due to the high sensitivity to photoperiod.

Major cultivated soybean varieties in high latitude areas will bloom early when planted in low latitudes, with short plants and few pods; whereas when varieties adapted to low latitude areas are planted in high latitude areas, they will bloom too late and can not complete their life cycles before the temperature drops in winter [83]. Different soybean cultivars exhibit distinct flowering time and maturity traits according to their habitats, and early maturity is usually accompanied by low yields. The response of soybean to photoperiod usually affects the length of the maturity period. Photoperiodic flowering regulation is an important agronomic trait, critical to soybean yield, quality and adaptability. Therefore, analysis of the molecular mechanism of soybean photoperiodic regulation of flowering can provide a theoretical basis for solving the contradiction between early maturity and high yield, and key core modules for the molecular design and breeding high-yield and high-quality soybean cultivation variety.

### 2.1. Molecular Basis of Soybean E Series Genes for Flowering-Time Regulation

As early as the 1920s, it was observed that soybean varieties sown in different times in a year bloom almost at the same time, so scientist used soybeans and tobacco (*Nicotiana tabacum*) as model plants to uncover the phenomenon of plant photoperiod [84]. According to the traditional approaches to genetics, a number of soybean major genetic variations have been found to be responsibile for wide adaptability of soybean plants. To date, a number of major genetic loci, namely *E1* [85], *E2* [86], *E3* [87], *E4* [88], *E5* [89], *E6* [90], *E7* [91], *E8* [92], *E9* [93], *E10* [94], *E11* [95], *J* [96] and several QTLs, such as *Tof11/Gp11* and *Tof12/Gp1/qFT12-1* [97], *Tof16* [98], *LJ16.1* and *LJ16.2* [99], have been identified to be involved in the control of flowering and maturity in soybean [83]. Dominant alleles of *E1*, *E2*, *E3*, *E4*, *E7*, *E8*, and *E10* inhibit flowering, whereas dominant alleles at *E6*, *E9*, *E11* and *J* promote flowering [83]. *E1*, *E3*, *E4*, *E7* and *E8* are involved in photoperiod sensitivity, especially to different light qualities under artificially induced LDs [100].

To date, *E1-E4* and *E9* have been cloned and studied in depth. *E4* and *E3* are phytochrome A (PHYA) genes, *GmPHYA2* and *GmPHYA3*, respectively. Soybean plants exhibit different responses to different red light: far-red light (R: FR) quantum ratios [101]. *GmPHYA3* (*E3*) participates in the control of flowering under LDs with a high R:FR quantum ratio [102], while *GmPHYA2* (*E4*) is responsible for the flowering response of LDs with a low R:FR ratio [91,101]. *E3* and *E4* double-dominant genotypes are sensitive to photoperiod response, which show delayed flowering and maturation, whereas homozygous double recessive genotypes are insensitive to photoperiod response, which are earlier flowering and maturation. *GmPHYA1* and *GmPHYA2* (*E4*) can coordinately regulate the photomorphogenesis under a low ratio of R: FR light [103]. *GmPHYA1* is likely to regulate the photoperiod sensitivity under LD conditions with a lower ratio of R: FR (<1.0) [104,105].

*E2* is a homolog of the *Arabidopsis GI* [106]. *E2* and its near-isogenic line (*e2*) display similar flowering times at high latitudes 43° N and mid-latitude 36° N [106], indicating that the regulation of soybean flowering period by *E2* may not depend on photoperiod, so it has a small impact on the photoperiod response. Studies have shown that the function of *E2* in soybean and that of *GI* in *Arabidopsis* may have been differentiated: the full-length *E2* gene is unable to rescue the late flowering phenotype of the *Arabidopsis gi* [107]. *E2* regulates flowering by inhibiting the transcription of *GmFT2a*, but the molecular mechanism is still unclear [20]. Based on studying of the *E2* haplotypes of cultivated soybeans and wild soybeans in different regions of China, it has been found that the diversification of *E2* haplotypes may help soybean flowering time adaptation, and this adaptation promotes the spread of domesticated soybeans [107].

Kong et al. discovered the *E9* gene [14], and the dominant *E9* gene confers early flowering, whereas the recessive *e9* gene confers late flowering [93]. *E9* is the soybean florigen gene *GmFT2a* [93,108]. The recessive *e9* gene is due to the retrotransposon SORE1 inserted into the first intron of the *GmFT2a* gene, which inhibits its function and delays flowering. Further study showed that both sides of the intron, where retrotransposon *SORE1* inserts, is methylated, and methylation affects the expression of *GmFT2a* gene [108]. Another *FT* homolog, *GmFT4* plays important roles in inhibiting soybean flowering under non-inductive LD conditions, and is strongly induced by LDs. *GmFT4* most likely is the candidate gene for the newly identified mature locus *E10* [94].

Soybean *E1* locus has the greatest impact on flowering and maturity periods, and is located near the centrioles of chromosome 6, which brings great difficulty to QTL mapping and cloning. Xia et al. found that E1 is a specific TF for legumes, containing binary nuclear localization signal, a DNA binding site, and a B3 domain. Mutations in the distant B3 domain reveal that this domain is closely related to the function of E1 to inhibit flowering [109]. The expression of *E1* is significantly inhibited under SD conditions, while a bimodal circadian pattern is exhibited under LD conditions, indicating that the *E1* gene was regulated by the photoperiod, and that LDs induce the expression of *E1*. This is the main reason for that soybean has become a SD crop. However, in the *e3/e4* genetic background, *E1* induction by LDs is eliminated, indicating that the *E1* gene is controlled by *E3* and *E4*. Soybean has two *E1* homologues, *E1La* and *E1Lb*, and their expression patterns are similar to *E1* under both LD and SD, *E1* and *E1L* genes repress flowering by down-regulating *GmFT2a* and *GmFT5a*, under LD conditions, but promote the expression of the flowering inhibitor *GmFT4* [110].

### 2.2. Core Components in the Photoperiod Pathway in Soybean

**Figure 2:**
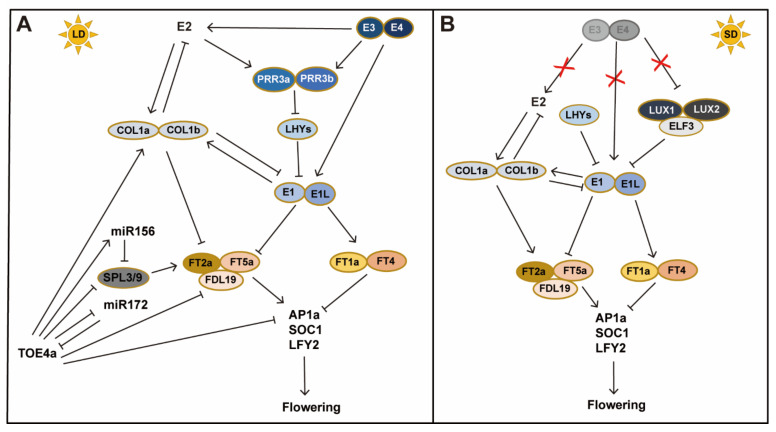
Working models for flowering-time regulation in soybean under long day (LD) and short day (SD). (**A**) Flowering-time regulation of soybean in LDs. Under LD, *E3* and E4 upregulate the expression of *E1*, *E1Lb*, *E2*, *GmPRR3a* and *GmPRR3b*. *E1* promotes the expression of the floral inhibitors *GmFT1a* and *GmFT4*, but inhibits the expression of the floral promoters *GmFT2a* and *GmFT5a*, and thus the transition to flowering is greatly delayed in LDs. GmFT2a and GmFT5a interact with GmFDL19 to induce the expression of *AP1a*, *SOC1* and *LFY*, whereas GmFT1a and GmFT4 function to inhibit the expression of these floral promoters to delay flowering. *GmPRR3a* and *GmPRR3b* repress *GmLHYs* expression through binding to their promoter. GmLHYs bind to the *E1* promoter to repress *E1* expression. The inhibition of *E1* expression by *GmLHY*s is released by the induction of *GmPRR3a* and *GmPRR3b*. *GmCOL1a* and *GmCOL1b* are soybean flowering inhibitors and repress *GmFT2a* and *GmFT5a* expression, and there is a feedback regulation among *GmCOL1a*, *E1* and *E2*. *E3* and *E4* promote the expression of *E2*. *GmTOE4*a delays flowering by inhibiting the expression of *GmFT2a*, *GmFT5a*, *GmSPL3/9* and *miR172*, and promoting the expression of *miR156*, *GmCOL1a* and *GmCOL1b*. *miR172* negatively feedback regulates its target gene *GmTOE4a*. *GmTOE4a* participates in the flowering-regulation pathway that requires *E3* and *E4*. (**B**) Flowering induction of soybean in SDs. Under SDs, *E3* and *E4* play a limited role, and *E1* expression is also suppressed; hence, the inhibition of *GmFT2a/GmFT5a* and the induction of *GmFT1a /GmFT4* by *E1* both are weakened; therefore, flowering is strongly promoted. The expression of *E1/E1L* is repressed by *GmLHY*s and *GmELF3*. *GmELF3* and *GmLHY*s act additively to control *E1* expression. *GmELF3* is suppressed by two PHYA proteins, E3 and E4. As the functions of E3 and E4 are greatly weakened under SDs, the inhibition of *GmELF3* is released. The GmELF3 protein interacts with GmLUX1 and GmLUX2 to form the evening complex of GmELF3-GmLUX1-GmLUX2. In short, the two molecular regulatory modules: *GmELF3/GmLUX1/GmLUX2 -E1/E1L-FT* and *GmLHYs-E1/E1L-FT*, function to induce soybean flowering in SDs.

The florigen gene *FT* and *FT* relatives are evolutionarily-conserved key flowering promoters. 12 *FT*-like genes have been identified in soybean [111]. Overexpression of *GmFT2a/2b*, *GmFT3a/3b* and *GmFT5a/5b* in *Arabidopsis*, promotes flowering, similar to the *Arabidopsis FT*; in contrast, overexpression of *GmFT1a/1b*, *GmFT4* and *GmFT6* delays flowering. In addition, *GmFT2d* has no function in cultivated and wild soybeans, and *GmFT2c* in some wild soybean accessions is functional and can promote flowering [112]. Various studies show that *GmFT2a* and *GmFT5a* are key floral integration factors in soybeans [85,106,111,113,114]. *GmFT2b* is homologous to *GmFT2a*, and *Gmft2b* mutants show delayed flowering only under LD conditions [115]. *GmFT2b* can influence the expression of *GmFT* genes, and *GmFT2a* and *GmFT5a* are significantly upregulated in *GmFT2b*-overexpression plants. *GmFT2b* haplotypes in different maturity groups play important roles in soybean variety distribution [115]. *GmFT1a* and *GmFT4* are up-regulated by *E1* and function as flowering inhibitors, and their overexpression inhibits the expression of the floral meristem identity genes *GmAP1b* and *GmAP1c*, and thus delays flowering [110,116]. *GmFT1a* and *FT4* exhibit inhibitory effects, suggesting that *FT*-like genes may have undergone functional differentiation in soybean, and the functional divergence most probably due to the differences in protein sequence and structure caused by changes in critical amino acids [117]. The expression of *GmFT2a/2b* and *GmFT5a* are induced in soybean leaves under inductive SDs, whereas the expression of *GmFT1a* and *GmFT4* are induced under LDs. *GmFT4* and *GmFT1a* are barely detectable under SD conditions [110,111,116], and there is no direct evidence to support that GmFT4 and GmFT1a are transported from leaves to SAM to inhibit soybean flowering. Although these two genes are highly expressed in leaves, they are also expressed in SAM at a low level [110,111,116]. Relative transcript abundance of the flowering promoters *GmFT2a/5a* and the flowering inhibitors *GmFT1a/4*, is important for determining the appropriate flowering time under different growth environments (Figure 2).

In the soybean photoperiodic response, the growth period genes *E1-E4* participate in photoperiodic regulation of flowering. *E3* has a greater effect on *E1* compared with *E4* [85,118,119]. *E1* and its family genes are all soybean flowering inhibitors, which strongly inhibit *GmFT2a* and *GmFT5a* and delay flowering [85,119]. GmFT2a and GmFT5a are the key flowering integration factors in soybeans, which coordinately regulate flowering in the photoperiod pathway [111]. *GmFT2a /5a* and *GmFT4* genes are all regulated by E1. Therefore, in the photoperiod response of soybean, there is a major flowering-regulation pathway, *E3/E4-E1-GmFT*. E1 is a unique TF for legume crops, so *E1* defines a unique regulatory pathway to control the flowering and maturity periods. 

There are several genes in the photoperiod flowering pathway to interact with *E1* in flowering regulation. Overexpression of *GmCOL1a*, an *Arabidopsis CO*-like gene, leads to delayed flowering under LDs, and the *Gmcol1b* mutant is early flowering. These indicate that soybean *GmCOL1a* and *GmCOL1b* are flowering inhibitors. When *GmCOL1a* is overexpressed, the soybean growth period genes *E1* and *E2* are down-regulated. However, *GmCOL1a* is up-regulated in the near-isogenic line of *E1* and *E2*, so it is very likely that there is feedback regulation among *GmCOL1a*, *E1* and *E2*. In addition, *E3* and *E4* promote the expression of *E2* and *GmCOL1a/1b* [118].

**Figure 3:**
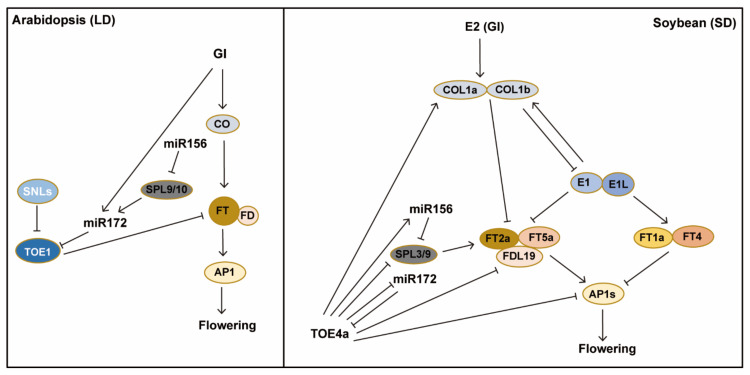
Comparison of photoperiodic flowering-regulatory pathways in *Arabidopsis* and soybean. The *GI-CO-FT* module is the core of photoperiodic regulation of flowering in *Arabidopsis*. Similarly, there is the *GI (E2)-CO-FT* module in soybean; in addition, there exists the *GI (E2)-CO-E1-FT* module in soybean. In *Arabidopsis*, GI regulates the expression of *CO*, *miR172* and *TOE1*, and miR172 regulates flowering through the module of GI-miR172-TOE1-FT, independent of CO; in addition, SPL9 and SPL10 directly activate *miR172* expression. In soybean, *GmTOE4a* is not regulated by the GI homolog E2, and there are two *GmTOE4a* modules: *TOE4a-miR156-SPL3/9-FT* and *TOE4a-mi172-FT*. Overexpression *TOE4a* represses the expression of *SPL3/9*. *GmFT2a/5a* promotes flowering, similar to the *FT/FD-AP1* module in *Arabidopsis,* whereas *FT1a/FT4* functions to repress soybean flowering.

The photoperiod response of soybean is an extremely complex process that requires the coordination of multiple genes to finally achieve flowering. miR172 and its target genes play a regulatory role in growth and development [120]. In the photoperiod-regulated soybean flowering network, *miR156* delays flowering, and the *E1* gene is also involved in the *miR156/GmSPL* pathway [121]. miR156 regulates its target gene *GmSPL3/9*, and miR172 act through its target gene *GmTOE4a* to feedback regulate of *miR156* and *GmSPL3/9*. There is also a negative feedback regulation between *miR172* and its target gene *GmTOE4a* [122]. Therefore, there may be a balance between *miR156* and *miR172*, which regulates soybean flowering at an appropriate time (Figure 3).

### 2.3. Molecular Mechanisms for Adaptation to Different Latitudes

High latitudes regions are associated with early winter and longer day lengths in summer, therefore, soybean needs to reduce photoperiod sensitivity to flower and mature early in LDs. *E1*, *E3*, and *E4* play an important role in defining photoperiod sensitivity and adaptation to high latitudes. Double recessive genotype *e3e4* is the most common genotype to reduce the photoperiod sensitivity, followed with *e1* and *e3* or *e4* [123]. *E1* shows functional redundancy with the *E1L* genes, and *e1* reduces but does not eliminate photoperiod insensitivity, but RNAi-mediated inhibition of *E1La* and *E1Lb* eliminates the residual flowering response [119]. There are dysfunctional and hypomorphic alleles of *E1*, *E3*, and *E4*, and usually are rare and region-specific [100,123,124]. Various combinations of the dysfunctional alleles at the *E3* and *E4* and *E1/E1L* loci, affect photoperiod sensitivity to varying degrees and help soybean adapt to longer day length in growth seasons at higher latitudes.

Recently, the QTLs of *Gp11/Tof11* and *qFT12-1/Gp12/Tof12*, which control soybean flowering and maturity time, have been identified. *Tof11* and *Tof12* encode homologs of *APRR3*[97,125,126], named as *GmPRR3a* and *GmPRR3b*, respectively. *Tof11* and *Tof12* are redundant to control flowering time. The two dominant *Tof11* and *Tof12* alleles flower much more later than the single dominant alleles in *Tof11* or *Tof12* [97,126], in line with the overexpression phenotype of the *Arabidopsis APRR3* [127]. *GmPRR3a* and *GmPRR3b* attenuate the inhibition of the circadian clock gene *LHY* on the legume-specific photoperiod regulation gene *E1*, leading to down-regulation of *GmFT2a/5a* and a delay in flowering. Overexpression of *GmPRR3a* and *GmPRR3b* increases the expression level of *E1* and decreases the expression of *GmFT2a* and *GmFT5a*. Loss of *GmPRR3a* and *GmPRR3b* function may allow early harvesting and improve adaptation to the limited summer growing season in high-latitude regions during soybean domestication. Population genetic analysis reveals that *Tof11* and *Tof1*2 have gradual mutations and are under artificial selection in breeding. The *tof12-1* mutation has been first strongly selected to accelerate the flowering and maturity periods of cultivars generally. The *tof11-1* mutation occurred after *tof12-1*, thereby further shortening the flowering period and growth period of cultivated soybeans, and thus improving the adaptability and planting of cultivated soybeans. Regardless of its origin, *tof12* exists in almost all improved soybean varieties [97,125,126], *GmPRR3a* (*tof11*) and *GmGIa* (*e2*) mutant alleles are also predominant in cultivated varieties of northern China and photoperiod-insensitive varieties [97,107,123], indicating that these three alleles have undergone strong artificial selection, and may provide the basis for the early stage of northward expansion.

*GmPRR37*, which encodes a pseudo-response regulator protein, is confirmed to be the major QTL *qFT12-2*, and *Gmprr37* mutants exhibit early flowering under LD conditions. Overexpression of *GmPRR37* significantly delays flowering, through down-regulated expression of *GmFT2a/5a* and up-regulated *GmFT1a* (a flowering-inhibitor). Further study shows that soybean adapts to higher latitudes by flowering early, relying in part on natural *GmPRR37* mutation [128].

A recent study shows that cotyledons facilitate the early-maturing soybean varieties adapting to high latitudes [129]. After the cotyledons emerged from the soil and exposed to light, *GmFT2a* was rapidly expressed at a high level, and the expression of downstream genes such as the floral meristem-identity gene *GmAP1* was up-regulated. This is an important mechanism for early-maturing soybean varieties to flower and mature early in high latitudes in LD conditions [129]. 

Low latitudes regions are associated with shorter day lengths in growth seasons, and thus it is necessary overcome the sensitivity to photoperiod when soybean is planted in these areas, otherwise, the flowering is too early and the yield will be significantly reduced. For example, before 1960, the soybean planting area in Brazil was limited to latitudes above 22° S, and soybean production was less than 10 million tons [83], until the long juvenile (LJ) traits were discovered and introduced in 1970s. The LJ trait plays an important role in soybean production in low-latitude regions, which allows the soybean flowering later and has an extended vegetative growth period under SDs. *E3* and *E4* play a minor role to control flowering under SD, as loss-of function *e3* and *e4* alleles have almost no effect on flowering under SD, compared to their respective wild-type alleles [130]. Some loci have been described, which may contribute to LJ, such as *E6* [90,131], *J* [130], *Lj16.1* and *Lj16.2* [132]. *LJ16.1* and *LJ16.2* encode the florigen (FT) homologs *GmFT2a* and *GmFT5a*, respectively. *Gmft2a* or *Gmft5a* single mutants show genetic compensation responses and have relatively little effect on flowering time, while the *Gmft2a Gmft5a* double mutations could break this compensation response, exhibiting enhanced *LJ* phenotype, so as to produce high yields in SD. *GmFT2a* and *GmFT5a* sequence diversity is a major factor in soybean spreading to lower latitudes [99].

Recently, the *J* gene has been cloned, encoding an ortholog of the *Arabidopsis ELF3* [130,133], and acting as a member of the circadian evening complex (EC). *E6* was also identified by map-based cloning. *E6* is a new allelic variant of the *J* gene, and the recessive *e6^PG^* allele contains a Ty1/copialike retrotransposon inserted in the fourth exon of *GmELF3* [134], so both *J* and *E6* are alleles of the *GmELF3* gene. *GmELF3* is suppressed by two PHYA proteins, E3 and E4 [130]. GmELF3 can bind to *E1* promoter regions and inhibits its expression under SDs. Recessive *j* alleles inhibit flowering under SD and prolong the vegetative period by releasing *E1* repression. Loss of *GmELF3* function results in a 30%–50% increase in yield [130]. Knockout of the two homologous *GmLUX1* and *GmLUX2*, encoding components of the EC complex, gives rise to a complete lost in photoperiod sensitivity. The *Gmlux1 Gmlux2* mutant shows a dramatically extended reproductive period, and thus the EC complex is the core of the soybean photoperiodic flowering control. The J protein interacts with GmLUX1 and GmLUX2 to form the soybean EC complex J-GmLUX1/GmLUX2, which binds to the promoters of *E1* and its two homologs *E1La* and *E1Lb* to suppress their expression, further relieving *GmFT2a* and *GmFT5a* expression and thus promoting flowering under SD conditions [135]. In *Arabidopsis*, mutants of EC components ELF3 and LUX have similar early-flowering phenotypes, indicating that the function of EC genes is conserved [136,137].

A novel locus *Time of Flowering 16* (*Tof16*), that delays flowering and improves yield at low latitudes, has been identified. *Tof16* encodes LHY1a, one of the four soybean homologs of the *Arabidopsis Circadian Clock Associated 1* (*CCA1*) and *Late Elongated Hypocotyl* (*LHY*) [98]. Under SDs, the loss of function allelic variation of *Tof16* significantly prolongs the flowering period and increases soybean yield. Tof16 directly regulates the expression of *E1*, and thus regulates the photoperiod flowering of soybean. There are four soybean *LHY* homologs to redundantly control flowering time and yield. Indeed, the quadruple knockout mutants produced by CRISPR-Cas9 shows significantly delayed flowering [98,138], whereas the *Arabidopsis lhy* mutant flowers early [139]. *Tof16* and *J* are independent in controlling the flowering period and yield of soybeans in low latitude regions, and have additive genetic effects. Further analysis revealed that more than 80% of soybean varieties in low-latitude regions bear loss-of-function allelic variants of these two genes, indicating that *Tof16* and *J* play very important roles in the adaptation of soybeans to low-latitude regions. Hence, natural variation at the *Tof16* or *J* locus is the main genetic basis for cultivated soybeans to adapt to tropical regions.

## 3. Conservation and Divergence of the Photoperiodic Regulation of Flowering in Soybean and *Arabidopsis*

The photoperiod pathways in soybean and *Arabidopsis* have both conservative and unique aspects (Table 1). In *Arabidopsis*, the circadian clock output gene *CO* plays an important regulatory role in photoperiodic regulation, and the photoperiodic regulation of flowering in *Arabidopsis* is the *GI-CO-FT* regulatory module (Figure 1). The function of *CO* in flowering regulation is relatively conserved, and usually plays a role in inducing flowering in many species such as *Arabidopsis*, and rice [140]. However, *GmCOL1a* and *GmCOL1b* act to repress flowering in soybean. This may be related to that *GmCOL1a/1b* expression is regulated by the soybean-specific TF E1. *GmCOL1a/1b* are regulated by the GI homologous gene *E2*, and function to repress the expression of *GmFT2a/5a*. Therefore, the photoperiod pathway is partially conserved in soybean, with the *GI (E2)-CO-FT* model, but the regulation of *GmCOL1a /1b* by *E1* is unique in soybean (Figure 2).

In *Arabidopsis*, GI not only regulates *CO* expression, but also regulates *miR17*2 and its target gene *TOE1* [53]. TOE1 binds to the *FT* promoter and inhibits *FT* expression in the morning [141]. There is the *GI-miR172-TOE1-FT* module to promote flowering in *Arabidopsis*. In soybean, *GmTOE4a* participates in the photoperiodic flowering regulation that requires *E3* and *E4*, which is dependent on the expression of *GmCOL1a*; however, *GmTOE4a* expression is not regulated by the GI homolog E2 [122]. This is different from the *miR172* pathway in *Arabidopsis*.

In *Arabidopsis*, the FT proteins move to the SAM to promote the expression of *SOC1*, *LFY*, *AP1* and other floral meristem identity genes, and thus induces flowering. In soybean, *GmFT2a* and *GmFT5a* have been confirmed by various studies as key floral promoters [111,114]. *GmFT2a* and *GmFT5a* redundantly control soybean flowering in response to day-length changes. Both GmFT2a and GmFT5a move from the leaves to SAM and interact with the soybean ortholog of the *Arabidopsis* bZIP transcription factor FD, GmFDL19 [113]. GmFT2a/5a-GmFDL19 directly binds to the *cis*-acting element of the *GmAP1a* promoter to promote the expression of *GmAP1a*, and other *Arabidopsis* orthologs of floral promoter genes, such as *SOC1* (*GmSOC1a* and *GmSOC1b*) and *LFY* (*GmLFY2*), thereby to induce flowering [113]. The *Gmap1* quadruple mutant shows late flowering phenotype under inductive SD conditions, whereas overexpression of *GmAP1a* results in early flowering [142]. These results indicate that, like *Arabidopsis*, the *FT/FD-AP1* module is highly conserved in soybeans. *GmFT2a/5a* have conserved ability to induce flowering, but the mechanisms underlying the regulation of these *FT* relatives are not well conserved between soybean and *Arabidopsis*.

Soybean photoperiod pathway starts with PhyA and is finally reflected in the changes in the expression level of *GmFTs*. The key core transcription factors in this pathway include E1 and its two homologs, E1La and E1Lb. E1/E1L inhibit the expression of *GmFT5a* and *GmFT2a*. Under inductive SDs, the expression of *E1* is inhibited by J (GmELF3) and Tof16 (GmLHYs) in soybean [98]. GmELF3 interacts with GmLUX1 and GmLUX2 to form the soybean evening complex of GmELF3-GmLUX1-GmLUX2. This complex functions as part of the circadian clock and directly binds to *cis*-acting elements in *E1/E1L* promoters, resulting in further relieving of the expression of *GmFT2a* and *GmFT5a*, and thus promoting floral transition [135]. Hence, the molecular pathway of *phyA (E3E4)-GmELF3/GmLHY-E1/E1L-FT*2a/5a induces rapid flowering in inductive SDs (Figure 3). Under LD conditions, the transition to flowering is greatly delayed in soybean. Long-day light exposure induces the expression of *GmPRR3a* and *GmPRR3b* in part through the two phytochrome A photoreceptors E3 and E4, leading to a partial release of the *E1* inhibition; hence, the molecular pathway of *phyA (E3E4)- GmPRR3a /3b-E1/E1L-FT2a/5a* eventually leads to flowering in LDs.

E1 is the core component in the soybean photoperiod pathway and functions to integrate the light signal and the circadian clock signal, which is similar to the role of *CO* in the long-day induction of flowering in *Arabidopsis*; however, *E1* and *CO* are not homologs. This highlights the divergence of photoperiodic regulation of flowering between the two eudicots *Arabidopsis* and soybean. Further research is needed to fully understand how day-length signals at different latitudes induce the transition to flowering and control seed maturation time to maximize seed production in soybean.

The molecular circuitry underlying photoperiodic regulation of flowering time in the SD plant soybean is only partially conserved in other SD plants. In rice, a facultative SD plant, the core components of photoperiod pathway including E2 (GI), CO and FT are evolutionarily conserved, but the *OsGI* (*GI* ortholog)-*OsHd1* (*Heading date 1*, *CO* ortholog)-*OsHd3* (*Heading date 3*, *FT* homolog) pathway functions differently from that in soybean in floral induction in response to SD signals [143,144,145,146]. The key player in soybean flowering regulation, E1, is not conserved in rice [85,143,147]. There are monocot-specific flowering regulators in rice and other grass plants; for instance, the potent floral repressor *Ghd7* (*Grain number, plant height and heading date 7*), which inhibits rice flowering in long days [148,149,150], is not found in *Arabidopsis* or soybean. Apparently, the molecular networks underlying photoperiodic regulation of flowering in different species possess species-specific characteristics for their adaptation to local seasonal day-length changes. This highlights the diversity and complexity of flowering-time regulation in diverse plants.

## Figures and Tables

**Table 1:** Comparison of photoperiodic flowering-regulatory genes in soybean and *Arabidopsis*.

Gene in Soybean	Function		*Arabidopsis* Ortholog	Function	
*E1*	Inhibition	[85]	None		
*E1La*	Inhibition	[119]	None		
*E1Lb*	Inhibition	[106,119]	None		
*GmGI* (*E2*)	Inhibition	[106]	*GI*	Promotion	[25,28]
*GmphyA3* (*E3*)	Inhibition	[102]	*PHYA*	Promotion	[5]
*GmphyA2* (*E4*)	Inhibition	[103]	*PHYA*	Promotion	[5]
*GmFT2a* (*E9*)	Promotion	[93,108,111]	*FT*	Promotion	[43,44]
*GmFT5a*	Promotion	[111]	*FT*	Promotion	[43,44]
*GmFT4* (*E10*)	Inhibition	[94,110]	*FT*	Promotion	[43,44]
*GmFT1a*	Inhibition	[116]	*FT*	Promotion	[43,44]
*GmCOL1a/1b*	Inhibition	[118]	*CO*	Promotion	[26]
*miR156*	Inhibition	[121]	*miR156*	Inhibition	[54,55]
*miR172*	Promotion	[122]	*miRNA172*	Promotion	[53,54]
*GmTOE4a*	Inhibition	[122]	*TOE1*	Inhibition	[7,141]
*GmPRR3a* (*Tof11*)	Inhibition	[97,125]	*APRR3*	Inhibition	[127]
*GmPRR3b* (*Tof12*)	Inhibition	[97,125]	*APRR3*	Inhibition	[127]
*GmELF3* (*J*)	Promotion	[130,133]	*ELF3*	Inhibition	[137]
*GmLUX1*	Promotion	[135]	*LUX*	Inhibition	[136]
*GmLUX2*	Promotion	[135]	*LUX*	Inhibition	[136]
*LHY1a (* *Tof16* *)*	Promotion	[98]	*LHY*	Inhibition	[139]
*LHY1b/c/d*	Promotion	[98]	*LHY*	Inhibition	[139]
*GmFDL19*	Promotion	[113]	*FD*	Promotion	[45]

“Inhibition” indicates floral inhibition; “promotion” for floral promotion.
